# The role of Emergency Departments for Antimicrobial stewardship in COVID-19 Pandemic; the time is now

**DOI:** 10.12669/pjms.38.1.5107

**Published:** 2022

**Authors:** Madiha Ismail, Nazeer Najeeb Kapadia, Sara Usman

**Affiliations:** 1 Dr. Madiha Ismail, FCPS. Department of Emergency Medicine, Aga Khan University Hospital, Karachi, Pakistan; 2 Dr. Nazeer Najeeb Kapadia, FCPS. Department of Emergency Medicine, Aga Khan University Hospital, Karachi, Pakistan; 3 Dr. Sara Usman, Department of Emergency Medicine, Aga Khan University Hospital, Karachi, Pakistan

Inadvertent use of antibiotics is a primary driver of antimicrobial resistance globally and posing a significant risk to patient safety. With scarce treatment options and considerable overlap of symptoms between COVID-19 and bacterial pneumonia, the reflex prescription of antibiotics has become a routine.^1^ The situation is even more alarming in lower-middle-income countries where multi-resistant organisms are rampant and over the counter prescription of restricted antibiotics is considered a norm; knowledge gaps of healthcare providers and lack of governmental policies and surveillance are equally at fault here.^2^

The WHO guidelines for the clinical management of COVID-19 advise clinicians to start empirical antimicrobial treatment only in severe cases. Bacterial co-infections are quite rare and reported as low as 3.5% internationally. Recent systematic reviews identified that 72% of patients with COVID-19 receive antibiotic therapy even though only 7% having a bacterial co-infection.^3^ In a case-control study from Pakistan, Nasir N et al. reported an overall antibiotic utilization at 82%; 64% in patients with no evidence of bacterial infection.^4^

In the developed world, systemic antibiotics related adverse events account for an estimated 142,500 ED visits each year. Emergency Department(ED) is an interface between the inpatient and community settings. The antibiotic choice made by the ED physician also influences the therapy continued in the inpatient setting. Also, ED plays a vital role in obtaining relevant cultures before administering antibiotics to tailor or stop antibiotic therapy during hospitalization. Globally, ED practitioners must acknowledge their role to address the increasing problem of antimicrobial resistance.^5^

Even if emergency care providers appreciate the public health implications of growing antimicrobial resistance, changing their practices in the ED is challenging due to frequent interruptions, high-volume care, the need for rapid decisions with limited information and time, variation in staff over different shifts and fear of patient’s dissatisfaction if not prescribed antibiotics.^6^ We propose an ED-inpatient integrated antibiotic stewardship program due to the anticipated rise in antibiotic resistance during the Covid-19 era. In addition, we call for developing locally relevant ASP guidelines, including targeted educative interventions for ED physicians on antimicrobial resistance, focusing on infection prevention and control, reserving antibiotics for critically ill Covid patients and most importantly PCT-based antibiotic review and downgrade in 48 hours.

## Authors Contribution:

**MI** conceived, wrote and edited the final manuscript.

**NNJ, SU** did the literature review and manuscript writing.
